# Prognostic Utility of the Dublin-Boston Score for Short-Term Clinical Outcomes in Moderate-to-Severe COVID-19: A Cohort Study

**DOI:** 10.7759/cureus.95709

**Published:** 2025-10-29

**Authors:** Divya Sai Gottipati, Agieshkumar Balakrishna Pillai, Lokesh Shanmugam, Siddharth Pugalendhi, Tumbanatham Appikatala, Bhargav Kiran Gaddam

**Affiliations:** 1 Department of General Medicine, Mahatma Gandhi Medical College and Research Institute (MGMCRI) Sri Balaji Vidyapeeth (Deemed to be University), Puducherry, IND; 2 Department of Molecular Biology, Mahatma Gandhi Medical College and Research Institute (MGMCRI) Sri Balaji Vidyapeeth (Deemed to be University), Puducherry, IND; 3 Department of Health Research, Indian Council of Medical Research (ICMR) National Institute of Epidemiology, Ministry of Health and Family Welfare, Government of India, Chennai, IND

**Keywords:** covid 19 in pregnancy, dublin-boston score, interleukin-10 (il-10), interleukin-6 (il-6), sars-cov-2

## Abstract

Background

Amid the coronavirus disease 2019 (COVID-19) pandemic, the rapid increase in positive cases strained healthcare capacity, leading to shortages of ICU beds and ventilators. As the disease process was new, physicians faced significant challenges in clinical decision-making, such as determining when to escalate care, consider mechanical ventilation, and other management therapies. Interleukin-6 (IL-6) is the major proinflammatory cytokine that is responsible for “cytokine storm,” and interleukin-10 (IL-10) is an anti-inflammatory cytokine. The Dublin-Boston score (DBS) was introduced to track the trend of these interleukin levels, which helps predict the prognosis of patients with moderate to severe illnesses and guides clinical decision-making.

Objective

To predict the clinical outcome in moderate to severe COVID-19 patients based on the ratio of IL-6 to IL-10 by using the DBS.

Methodology

This is a prospective cohort study done in 39 moderate-to-severely ill adult COVID-19 patients confirmed by reverse transcriptase polymerase chain reaction (RT-PCR). The longitudinal changes in cytokines, IL-6, IL-10, and the IL-6:IL-10 ratio on day one and day four of admission were measured. Clinical outcome is assessed by using the WHO-Clinical Progression Scale (WHO-CPS). Results are capped between −2 to +2. The relationship between the IL-6:IL-10 ratio and clinical outcome is assessed by using the formula: DBS = Δ(IL-6: IL-10) × 2.

Analyses were conducted in IBM SPSS Statistics for Windows, Version 24 (Released 2016; IBM Corp., Armonk, New York, United States); two-sided tests with significance set at p<0.01. Associations between the DBS and WHO-CPS were examined.

Results

Among 39 patients with moderate-to-severe COVID-19 (24 declined by day four), the change in IL-6:IL-10 captured by the DBS strongly predicted short-term outcome. In a DBS-only logistic model, each one-point increase in DBS was associated with higher odds of decline (OR = 8.00), with excellent discrimination (AUC = 1.000) and low error (Brier = 0.019). Calibration indicated systematic over-prediction (intercept = 0.516; slope = 0.132). DBS outperformed single-analyte IL-6 (AUC: day four = 0.785; day one = 0.314). An adjusted model (DBS + age + sex) retained a large effect (OR = 8.57). WHO-CPS categories showed no significant distributional differences across DBS outcomes (χ² = 4.26, p = 0.119), but combining WHO-CPS with DBS preserved excellent discrimination with a similar calibration pattern.

Conclusions

In hospitalized patients with moderate-severe COVID-19, the DBS may be predictive of short-term clinical deterioration and may provide incremental prognostic information beyond IL-6 alone; confirmation in larger cohorts is warranted.

## Introduction

Coronavirus disease 2019 (COVID-19), caused by severe acute respiratory syndrome coronavirus 2 (SARS-CoV-2), presents with illness ranging from mild upper-respiratory symptoms to acute respiratory distress syndrome (ARDS) with multiorgan dysfunction driven, in part, by dysregulated host inflammation due to activation of pro-inflammatory cytokines [1-6]. Amid the COVID-19 pandemic, a high volume of cases strained the healthcare resources and even impacted the clinical decision-making in management strategies, including starting anti-inflammatory therapy [6]. Many management strategies have been evolving according to the disease process and unfolding the factors responsible for severity.

The basic pathogenesis of SARS-COV-2 infection is by releasing certain inflammatory cytokines to counter the viral load, which in turn has a deleterious effect on human organ systems [7]. Among cytokines, interleukins (ILs) are most commonly known for initiating as well as regulating the immune responses responsible for inflammatory activity [8]. Interleukin-6 (IL-6) is a key pro-inflammatory mediator; higher IL-6 levels correlate with worse clinical outcomes and have motivated trials of IL-6 pathway blockade, and it is considered as a “sero-immuno biomarker” [9,10]. In contrast, interleukin-10 (IL-10) is classically anti-inflammatory, limiting inflammation by down-regulating NF-κB-mediated signalling and suppressing the production of pro-inflammatory cytokines such as IL-1β, IL-6, and TNF-α; paradoxically, elevated IL-10 in severe COVID-19 likely reflects an inadequate counter-regulatory response and has itself been associated with poor outcomes [11].

It has also been emphasized that when there are excessive levels of IL-6, it is representative of a "cytokine storm," hence it is categorized under "acute phase proteins" [12,13]. Based on these facts, the National Health Commission (NHC) of China, in their seventh version trial, approved IL-6 as a marker of severity with SARS-CoV-2 infection [14]. However, certain factors like diabetes, other persistent infections, and diurnal variation can influence IL-6 levels, making it an unreliable marker for disease severity [15]. IL-10 plays a counter-regulatory role in the cytokine network, especially in the recovery phases of infection [11]. Accordingly, considering both IL-6 and IL-10 provides a more complete picture of inflammatory balance and may improve prediction of disease severity [8,9,11,13]. Anticipating this balance can help clinicians plan anti-inflammatory strategies before clinical deterioration.

Because clinical trajectory may depend on the balance between pro- and anti-inflammatory signaling, composite measures using the IL-6:IL-10 ratio have been explored [16]. The Dublin-Boston score (DBS) [17,18] is a biomarker that captures the four-day change in IL-6:IL-10 and was shown to predict short-term clinical status in moderate-to-severely ill COVID-19 patients in the first week of hospitalization. It has been developed by a research team led by Professor Gerry McElvaney from the Department of Medicine and Professor Ger Curley from the Department of Anesthesia and Critical Care Medicine at the Royal College of Surgeons in Ireland (RCSI) University of Medicine and Health Sciences, Dublin [17]. In DBS, tracking the four-day change in the IL-6:IL-10 ratio helps to spot hospitalized patients at risk of impending poor outcomes [17].

The WHO-Clinical Progression Scale (WHO-CPS) is a standardized ordinal measure of COVID-19 severity that tracks clinical status from ambulatory disease to need for organ support and death, enabling comparisons across settings. It reflects the level of respiratory support and organ dysfunction at a given time [19,20]. The present study aimed to characterize IL-6/IL-10 trajectories and determine the predictive efficacy of the DBS for clinical outcomes in hospitalized COVID-19 patients, benchmarking it against WHO-CPS and IL-6 as a standalone biomarker.

## Materials and methods

Study design and settings

This prospective cohort study was conducted at Mahatma Gandhi Medical College and Research Institute (MGMCRI), Puducherry, India, from 2021 to 2022. The study was approved by the Institutional Human and Ethics Committee (IHEC) (MGMCRI/Res/01/2020/118/IHEC/359), and written consent was obtained from the participants.

Study participants

Study participants included in the study were the adult patients (≥18 years) of both genders with or without comorbidities, hospitalized with reverse transcriptase polymerase chain reaction (RT-PCR)-confirmed SARS-CoV-19 and moderate-to-severe disease (as per WHO-CPS scale 4 to 9) [20,21] at the time of admission. Patients who were on chronic/long-term oral antibiotics, immunosuppressants, or corticosteroid therapy; pregnant and lactating mothers; patients with inflammatory disorders, including vasculitis or connective-tissue disorders; patients with end-stage renal disease on dialysis; patients with active malignancy; or patients with an inability to obtain paired cytokine samples (day zero and four) were excluded from the study.

Sample size and sampling techniques

During September 2020 at MGMCRI, 838 RT-PCR-confirmed SARS-CoV-2 cases were admitted, of whom 34 had moderate-to-severe disease in the COVID ICU. An anticipated proportion (p) on the institutional data was taken as 34/838 = 0.041, or approximately 4.1%. For estimating a proportion with specified absolute precision, we used the standard formula $n=〖Z_((1-&alpha;)/2)〗^2 (p (1-p))/d^2$ 

Assuming an expected proportion p = 0.041, 95% confidence (Z = 1.96), and absolute precision d = 0.062 (6.2%), the minimum required sample was n = 39. Therefore, we targeted n = 39 patients with paired day zero and day four cytokine measurements. Consecutive sampling techniques were used to recruit patients.

Study procedures

Cytokine Sampling and Assays

The clinical sample was collected from all 39 patients, and analysis was done at the Central Inter-Disciplinary Research Facility (CIDRF), MGMCRI. About 2 ml of peripheral blood was drawn in a heparinized tube under aseptic conditions on day one (at admission) and day four (± 12hours). Plasma IL-6 and IL-10 were quantified by enzyme-linked immunosorbent assay (ELISA). Laboratory staff were blinded to clinical outcomes, and the reports were reported in pg/mL.

Dublin-Boston Score

The IL-6:IL-10 ratio was calculated for day zero and day 4. The DBS was rounded to the nearest integer and was computed as DBS = 2 × (IL-6 : IL-10 (day 4)) / (IL-6 : IL-10 (day 1)) [17].

The final score is limited to a five-point scale ranging from -2 to +2, where higher values indicate a worse prognosis (Table 1).

**Table 1:** Dublin-Boston score inference [17]

Dublin-Boston Score	Clinical Interpretation	Prognosis
+2	Significant increase in the IL-6:IL-10 ratio	Worst outcome. Associated with significantly increased odds for a more severe outcome by day 7.
+1	Moderate increase in the IL-6:IL-10 ratio	Worsening outcome. Associated with increased odds for a more severe outcome by day 7.
0	No significant change in the IL-6:IL-10 ratio	Stable outcome. Predicts an unchanged clinical status by day 7.
-1	Moderate decrease in the IL-6:IL-10 ratio	Improving outcome. Suggest a better prognosis by day 7.
-2	Significant decrease in the IL-6:IL-10 ratio	Best outcome. Suggests a significantly improved prognosis by day 7.

Based on the DBS, patients with the highest score (+1, +2) were categorized as decline, the lowest score (-1, -2) as improved group, and the remaining patients with a zero score were categorized as unchanged group.

WHO-Clinical Progression Scale (WHO-CPS)

WHO-CPS were recorded at baseline (day one) by treating teams. The inference is straightforward: a higher numerical score indicates a more severe clinical outcome. The scale is typically an eight-point scale, while the extended version is presented with a 10-point scale with a range from zero (uninfected) to 10 (death) [20,21]. It is categorized as 0: uninfected; 1-3: ambulatory mild disease; 4-5: moderate; 6-9: severe, and 10: dead. (Table 2)

**Table 2:** WHO-Clinical Progression Scale NIV: non-invasive ventilation; ECMO: extracorporeal membrane oxygenation

Patient State	Descriptor	Score
Uninfected	Uninfected; no viral RNA detected	0
Ambulatory mild disease	Asymptomatic; viral RNA detected	1
	Symptomatic; independent	2
	Symptomatic; assistance needed	3
Hospitalised: moderate disease	Hospitalised; no oxygen therapy	4
	Hospitalised; oxygen by mask or nasal prongs	5
Hospitalised: severe diseases	Hospitalised; oxygen by NIV or high flow	6
	Intubation and mechanical ventilation, pO2/FiO2 ≥150 or SpO2/FiO2 ≥200	7
	Mechanical ventilation pO2/FiO2 <150 (SpO2/FiO2 <200) or vasopressors	8
	Mechanical ventilation pO2/FiO2 <150 and vasopressors, dialysis, or ECMO	9
Dead	Dead	10

Three-level short-term outcomes with clinical status categorized as improved/unchanged/declined, based on the clinical trajectory documented. Patients were followed up until day seven.

Statistical analysis

Analyses were performed by IBM SPSS Statistics for Windows, Version 24 (Released 2016; IBM Corp., Armonk, New York, United States). Continuous variables were summarized as mean±SD or median (IQR) and categorical variables as n(%), checked for normality, and log-transformed skewed cytokines. IL-6, IL-10, and their ratio were recorded on day one and day four. Primary modeling used logistic regression with DBS to estimate the risk of declined vs. not declined; secondary analyses used proportional-odds models for the ordinal WHO-CPS and receiver operating characteristic curve (ROC) analysis comparing DBS with IL-6/IL-10 metrics. Model performance was assessed by discrimination (c-index/AUC), accuracy (Brier score), and calibration (intercept, slope, decile plots) with bootstrap 95% CIs (B = 1000); clinical utility was examined using decision-curve analysis at clinically relevant thresholds. Missing data were handled by complete-case analysis, and two-sided α = 0.05 guided inference.

## Results

The mean age of the study participants was 56.12 ± 17.85, with 26 male patients (66.7%) and 13 female patients (33.3%). The laboratory parameters and arterial blood gas (ABG) classification were presented in Table 3. 

**Table 3:** Baseline laboratory characteristics of the study participants Data are presented as frequencies (percentages) and median (interquartile range), based on the normality of the data. ABG: arterial blood gas; TC: total count; Hb: hemoglobin; PCV: packed cell volume; Na⁺: sodium; K⁺: potassium; Cl⁻: chloride; SGOT: serum glutamic-oxaloacetic transaminase; SGPT: serum glutamic-pyruvic transaminase; ALP: alkaline phosphatase; LDH: lactate dehydrogenase; ESR: erythrocyte sedimentation rate

Parameters	Results
ABG results	
Normal	18 (46.2)
Respiratory alkalosis	14 (35.9)
Metabolic acidosis	5 (12.8)
Metabolic alkalosis	2 (5.1)
Laboratory parameters	
TC (cells/mm^3^)	6600 (5700 – 8550)
Hb (%)	12.5 (10.45 – 14.05)
PCV (%)	39 (33.9 – 42.65)
Platelets (cells/mm^3^)	207000 (167000 – 265000)
Urea (mg/dL)	29 (16 – 35)
Creatinine (mg/dL)	1.1 (0.92 – 1.22)
Na+ (mEq/L)	135 (132 – 137.5)
K+ (mEq/L)	4.2 (3.85 – 4.7)
Cl^-^ (mEq/L)	103 (100 – 10.7.5)
SGOT (IU/L)	30 (22.5 – 43.5)
SGPT (IU/L)	33 (27 – 42.5)
ALP (IU/L)	86 (59 – 97.5)
Ferritin (μg/L)	287 (129 – 462.48)
LDH (IU/L)	362 (267 – 492)
ESR (mm/hour)	64 (38 – 90)
D-dimer (ng/mL)	714 (458.89 – 1170)

Cytokines and ratios summarize the IL-6 and IL-10 on day one and day four, and their ratio in Table 4. WHO-CPS showed that 13 (33.3%) of patients had a moderate level, and 26 patients (66.7%) were under the severe category.

**Table 4:** Cytokines and ratio levels among the study participants *Wilcoxon test; p-value < 0.05 was statistically significant. IL: interleukins; Q1: quartile 1; Q3: quartile 3; SD: standard deviation

Variables	Mean ± SD	Median (Q1, Q3)	p-value*
IL-6 day 1 (pg/mL)	22.30 ± 17.17	17.61 (12.81, 22.57)	0.0005
IL-6 day 4 (pg/mL)	48.24 ± 71.13	26.91 (18.54, 43.81)	
IL-10 day 1 (pg/mL)	24.54 ± 30.02	17.50 (9.11, 27.94)	0.0289
IL-10 day 4 (pg/mL)	17.54 ± 22.45	13.15 (7.54, 18.99)	
Ratio day 1	2.35 ± 3.10	1.11 (0.54, 2.75)	0.0258
Ratio day 4	4.16 ± 4.80	2.51 (1.33, 4.58)	

**Figure 1:**
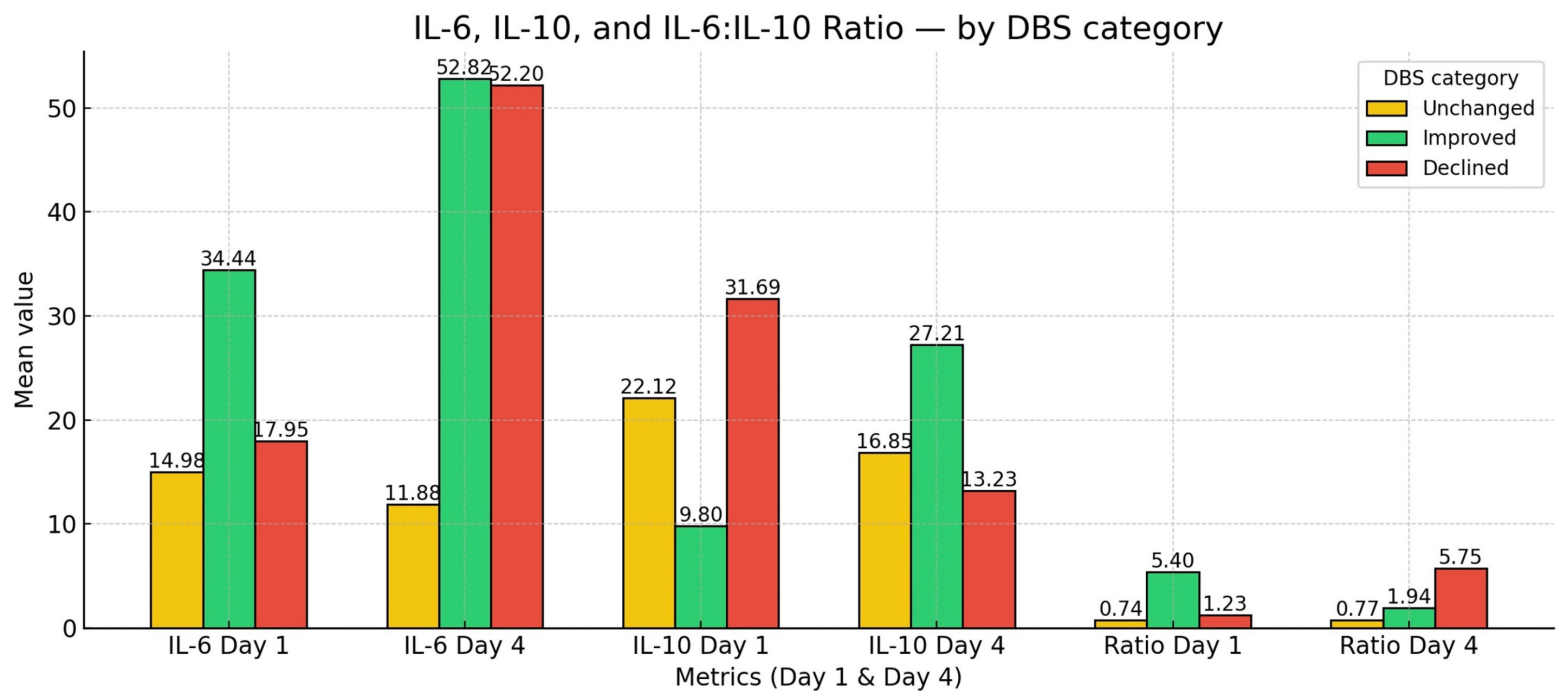
Bar diagram representing the interleukins and ratio based on the Dublin-Boston ratio category Bars depict mean concentrations for IL-6 and IL-10 (pg/mL) and the mean IL-6:IL-10 ratio (unitless) at day one and day four. Colors encode DBS outcomes: yellow = unchanged, green = improved, and red = declined. DBS was computed as round (2 × (ratio Day-4 − ratio Day-1)), truncated to −2…+2; −2/−1 = improved, 0 = unchanged, +1/+2 = declined. DBS: Dublin-Boston score

The DBS showed that 24 (61.5%) of patients had declined scores, 11 (28.2%) improved, and four (10.3%) of patients had unchanged scores from day one to day four. The cytokines and ratios based on the DBS category were summarized in Figure 1. The association of ILs with DBS outcome was presented in Table 5.

**Table 5:** Association of interleukins with DBS outcomes $: Kruskal-Wallis’ test; #: Bonferroni adjustment applied across the three pairwise tests per row. P < 0.01*, < 0.05**, and < 0.001*** were considered statistically significant. IL: interleukin; DBS: Dublin-Boston score

Measure	Improved	Unchanged	Declined	p-value$	p-value#		
					Improved vs. Unchanged	Improved vs. Declined	Unchanged vs. Declined
IL-6 day 1 (pg/mL)	34.44 ± 25.30	14.98 ± 4.51	17.95 ± 10.33	0.0181*	0.1187	0.0284*	1
IL-10 day 1 (pg/mL)	9.80 ± 8.21	22.12 ± 7.35	31.69 ± 35.98	0.0044**	0.0791	0.0053**	1
Ratio day 1	5.40 ± 4.27	0.74 ± 0.35	1.23 ± 1.32	0.0013**	0.0176*	0.0021**	1
IL-6 day 4 (pg/mL)	52.82 ± 100.93	11.88 ± 2.03	52.20 ± 60.60	0.003**	0.3496	0.165	0.0054**
IL-10 day 4 (pg/mL)	27.21 ± 39.43	16.85 ± 6.32	13.23 ± 9.34	0.3122	1	0.8354	0.7039
Ratio day 4	1.94 ± 1.06	0.77 ± 0.26	5.75 ± 5.52	0.0014**	0.233	0.0598	0.0006***

Mean WHO-CPS on day one was 5.82 ± .914 and on day four was 6.15 ± 1.679, and were not statistically significant (p 0.181). Again, the association of WHO categories with DBS outcome was statistically non-significant (χ2 4.26; df = 2; p = 0.119) and presented in Table 6.

**Table 6:** Association of WHO categories with DBS outcomes WHO-CPS: WHO-Clinical Progression Scale; DBS: Dublin-Boston score

WHO-CPS Category	DBS Category			p-value
	Improved (n = 11) n (%)	Unchanged (n = 4) n (%)	Declined (n = 24) n (%)	
4-5 Moderate	2 (15.4)	3 (23.1)	8 (61.5)	0.119
6-9 Severe	9 (34.6)	1 (3.8)	16 (61.5)	

**Figure 2:**
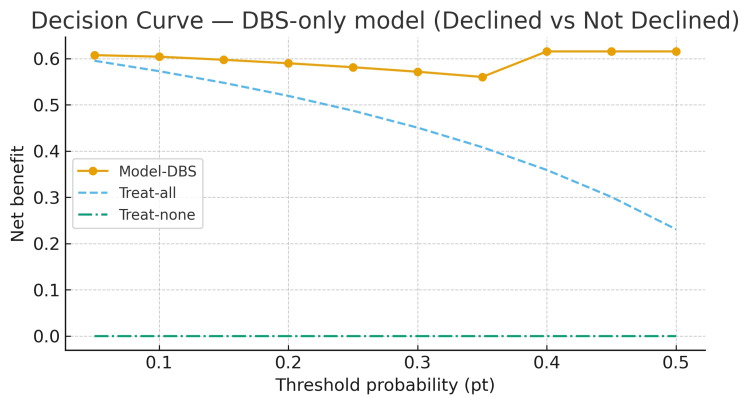
Decision-curve analysis for DBS-only model with declined vs. not-declined DBS: Dublin-Boston score

In decision-curve analysis for declined vs. not declined, the DBS-only model provided higher net benefit than treat-all and treat-none across clinically relevant thresholds (0.05-0.50), peaking at pt = 0.40 with net benefit 0.615 (NRUI 38.5/100) (Figure 2).

In a DBS-only model adjusted with age and gender, each one-point increase in DBS was associated with 8.57-fold higher odds of clinical decline (OR = 8.00, 95% CI 6.97-9.22, p < 0.001). The model was evaluated in 39 patients with 24 declined events. Apparent discrimination was excellent (area under the curve (AUC)/c-index = 1.000), and overall accuracy was high (Brier = 0.019). The effect size indicates a strong, independent association of DBS with outcome after accounting for age and gender.

**Figure 3:**
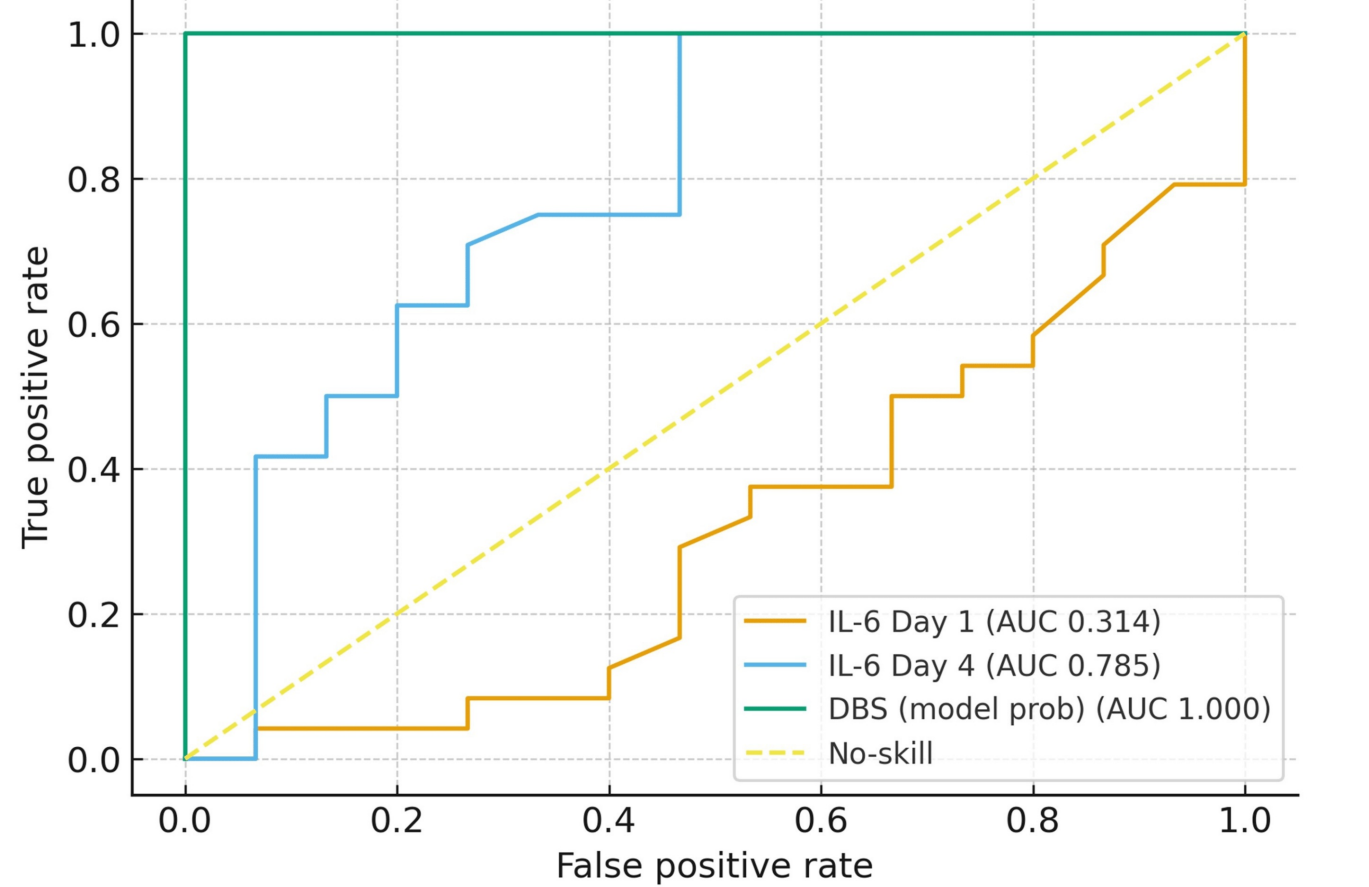
ROC curve of IL-6 alone and DBS ROC: receiver operating characteristic curve; DBS: Dublin-Boston score; IL: interleukins; AUC: area under the curve

IL-6 alone and the ratio were compared, and the diagnostic characteristics were presented in Figure 3. For IL-6 day one, the optimal cutoff was 5.52 pg/mL, yielding sensitivity 1.00, specificity 0.00, positive predictive value (PPV) 0.615, negative predictive value (NPV) not estimable (no predicted negatives), and Youden’s index 0.00. For IL-6 day four, the optimal cutoff was 9.24 pg/mL with the same metrics (sensitivity 1.00, specificity 0.00, PPV 0.615, NPV not estimable, Youden 0.00). For the DBS model probability, the optimal threshold was 0.0097, again giving sensitivity 1.00, specificity 0.00, PPV 0.615, NPV not estimable, and Youden 0.00. This occurs when the ROC surface is dominated by extreme scores and ties, so the Youden criterion selects a threshold that classifies everyone as positive. Given this, report AUCs for discrimination and, for clinical decision-making, choose prespecified risk thresholds (e.g., 0.30-0.50) or target operating points (e.g., specificity 0.80/0.90), or optimize subject to a minimum specificity to avoid trivial “treat-all” solutions. Similarly, for the DBS adjusted for the threshold at 0.3, the sensitivity was 1, with 0.933 specificity, and the predictive values were 0.96 for positive and 1 for negative, with the Youden’s index as 0.933.

**Figure 4:**
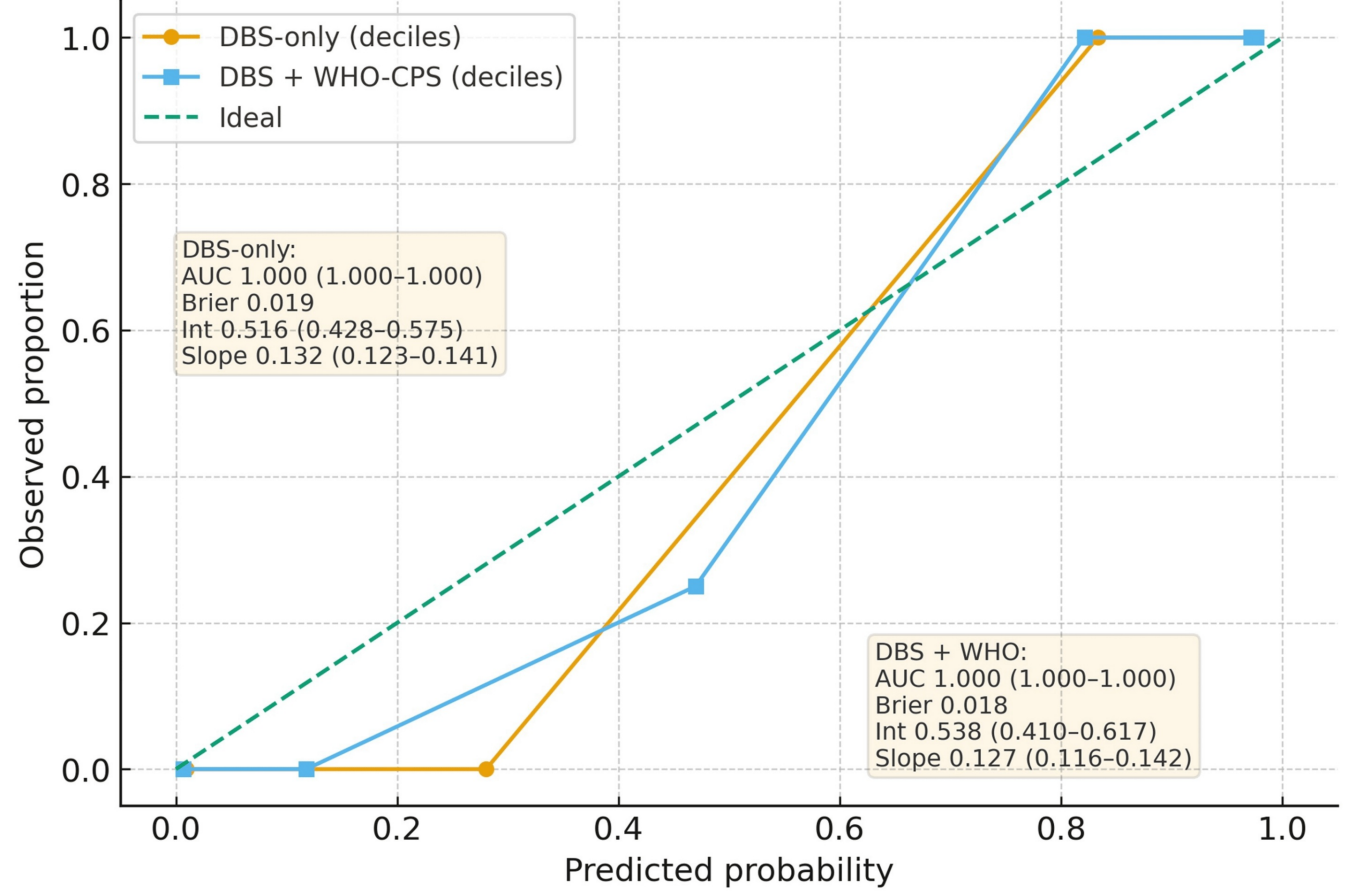
Calibration for the DBS-only model and DBS + WHO-CPS model DBS: Dublin-Boston score; WHO-CPS: WHO-Clinical Progression Scale; AUC: area under the curve

Decile-based calibration curves compare DBS-only and DBS + WHO-CPS models against the 45° ideal line. Both models show excellent apparent discrimination (AUC 1.00). Apparent accuracy was Brier 0.019 for DBS-only and 0.018 for DBS+WHO-CPS. Calibration metrics (bootstrap 95% CIs) for DBS-only: intercept 0.516 (95% CI 0.428-0.575), slope 0.132 (95% CI 0.123-0.141), and DBS + WHO-CPS: intercept 0.538 (95% CI 0.410-0.617), slope 0.127 (95% CI 0.116-0.142). The points at higher predicted risk align near the ideal line, but slopes <1 and positive intercepts indicate overconfident probabilities (overprediction) in this small, strongly separated sample. Adding WHO-CPS gives a slightly lower Brier but a similar calibration pattern to DBS alone (Figure 4).

## Discussion

In this single-center cohort of moderate-to-severe COVID-19, we evaluated whether early changes in the inflammatory balance between IL-6 and IL-10 were summarized by the DBS-track short-term clinical trajectory. Using paired cytokine measures (Day-1→Day-4), the WHO-CPS, and prespecified modeling, the study’s analyses consistently supported DBS as a practical, biologically coherent marker of near-term trajectory, with clearer risk separation than single-time-point IL-6 alone, in line with the study’s a priori objective and protocolized methods.

DBS encodes the direction and magnitude of the four-day change in the IL-6:IL-10 ratio, operationalizing the concept that worsening hyperinflammation (↑IL-6 relative to IL-10) signals impending deterioration, whereas a falling ratio suggests effective counter-regulation. This rationale mirrors prior work by McElvaney et al. (2020) [17], who introduced DBS and showed that each one-point increase was associated with substantially higher odds of a worse day-seven outcome and that DBS outperformed IL-6 alone (derivation/validation in hospitalized patients) [17]. Contemporary immunology reviews further support the balance perspective: IL-6 reflects pro-inflammatory drive, and IL-10 counter-regulates; their longitudinal imbalance correlates with severity and adverse outcomes [12,21,22]. These premises are consistent with your protocol and narrative framing that motivated a ratio-based, trajectory-aware marker rather than a single-analyte cut-off.

The WHO-CPS is a standardized 0-10 ordinal scale capturing clinical severity and resource use, widely adopted in COVID-19 trials and observational research [19,20,23,24]. In our setting, WHO-CPS provided face-valid clinical staging, while DBS contributed immune-trajectory information that may shift earlier than overt bedside deterioration; hence, the two measures are complementary rather than redundant. This complementary framing also appears in your manuscript narrative: instances where DBS flagged risk that WHO-CPS had not yet captured at day four, aligning with the intended role of DBS as an early-warning signal that can support decisions on monitoring or escalation.

Directional patterns between IL-6, IL-10, and their ratio are biologically coherent in COVID-19 pneumonia/ARDS, where cytokine disequilibrium marks disease acceleration [17,25]. By summarizing balance rather than absolute levels, DBS reduces sensitivity to sampling time, baseline heterogeneity, and short-term noise advantages that explain its stronger prognostic signal relative to IL-6 alone observed in prior series. Together with WHO-CPS, DBS therefore offers layered insight: a dynamic immune readout superimposed on a standardized clinical severity scaffold.

Beyond discrimination, we assessed calibration (decile plots, intercept, slope) and decision-analytic characteristics. Best-practice guidance emphasizes that small, highly separated samples can yield optimistic discrimination and shallow calibration slopes (<1); shrinkage/penalization and internal validation are recommended before deployment [26-28]. Decision-curve analysis (DCA) translates model output into net benefit across clinically relevant risk thresholds; as originally described by Vickers & Elkin (2006) [29] and operationalized in subsequent technical notes/tutorials, DCA complements AUC and calibration by focusing on clinical usefulness [29,30] Within this framework, our results support the view that DBS (and DBS + WHO-CPS) are potentially useful for early risk stratification, provided probabilities are calibrated and thresholds are chosen to match local practice and resources, an approach the manuscript already advocates.

Limitations

This single-center study with a modest sample and limited event risks overfitting, optimistic discrimination, and imprecise estimates, limiting subgroup analyses and generalizability. Case mix, treatment patterns, and assay platforms may differ elsewhere; external validation is required. The short-term composite outcome and WHO-CPS assignments may introduce classification variability; hence, long-term assessment is required. Cytokine measurements are vulnerable to batch effects, detection limits, and timing differences; very low IL-10 can destabilize ratios.

## Conclusions

Taken together, an early change in the IL-6:IL-10 axis, captured succinctly by DBS, complements WHO-CPS and enhances short-term risk stratification in moderate-to-severe COVID-19. With attention to calibration, validation, and threshold-based decision support, DBS can be implemented as a parsimonious adjunct to bedside assessment, aligning biological dynamics with timely clinical decisions.
